# Supplementary figures and images for: 60S dynamic state of bacterial ribosome is fixed by yeast mitochondrial initiation factor 3
Source: PeerJ. 2018 Sep 17;6:e5620. doi: 10.7717/peerj.5620 (PMC6147165; doi:10.7717/peerj.5620)

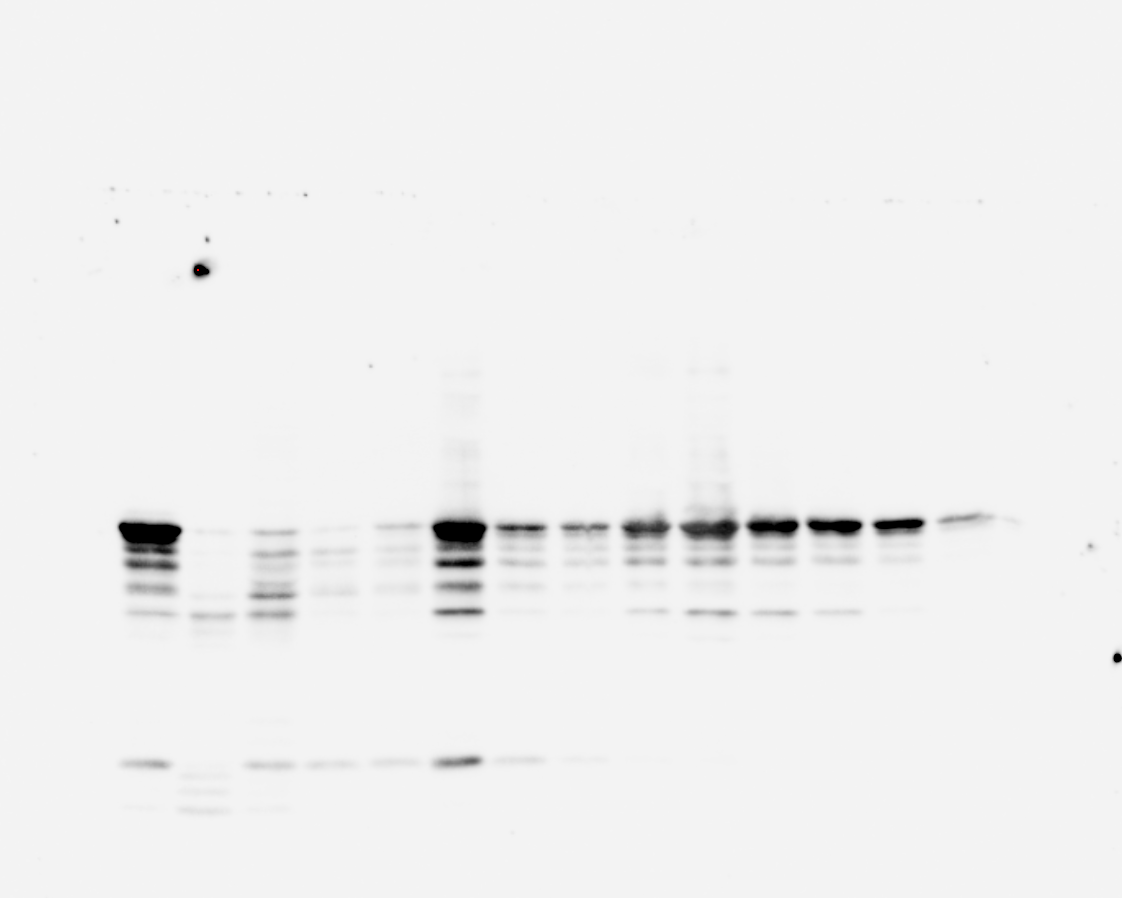

Supplement: Supplemental Information 3 — Non-modified Western-blots. [file peerj-06-5620-s003.zip › Fig2B Aim23 raw data.tif]

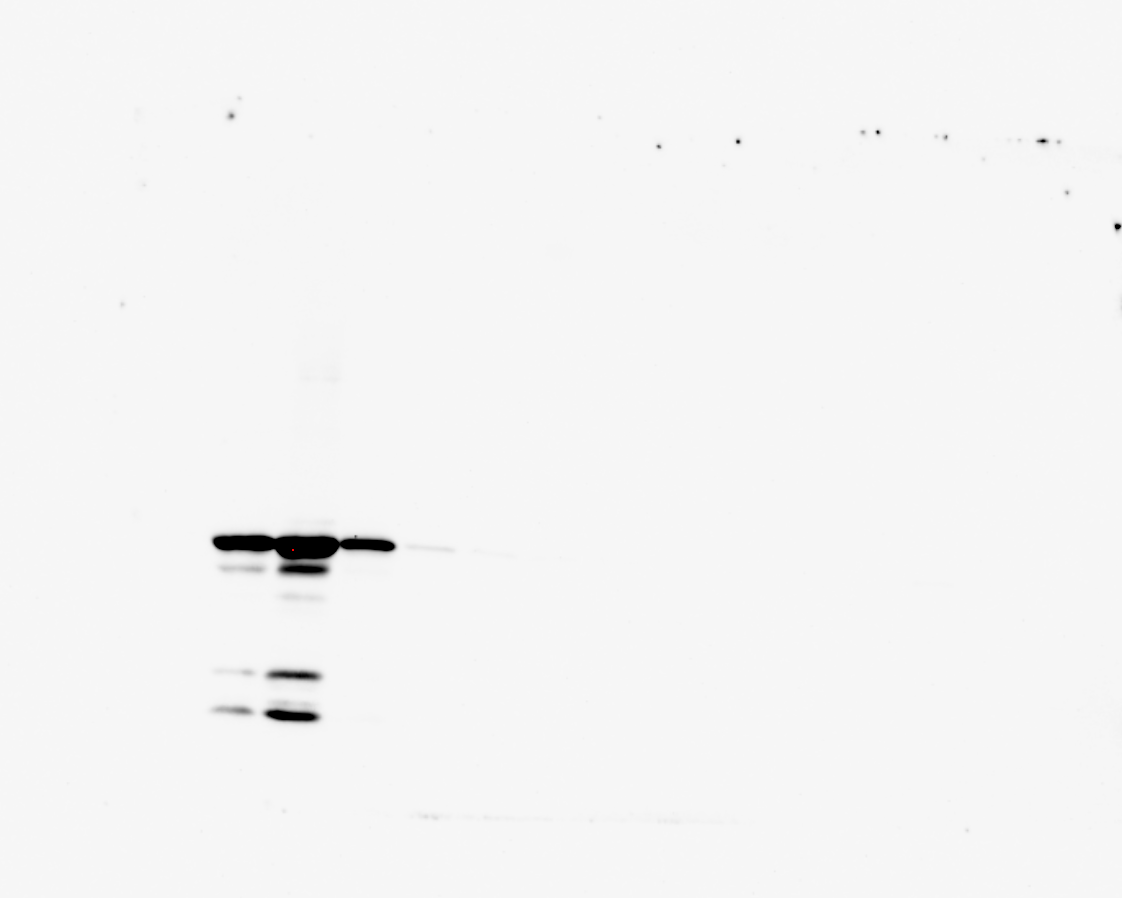

Supplement: Supplemental Information 3 — Non-modified Western-blots. [file peerj-06-5620-s003.zip › Fig2B Aim23dd raw data.tif]

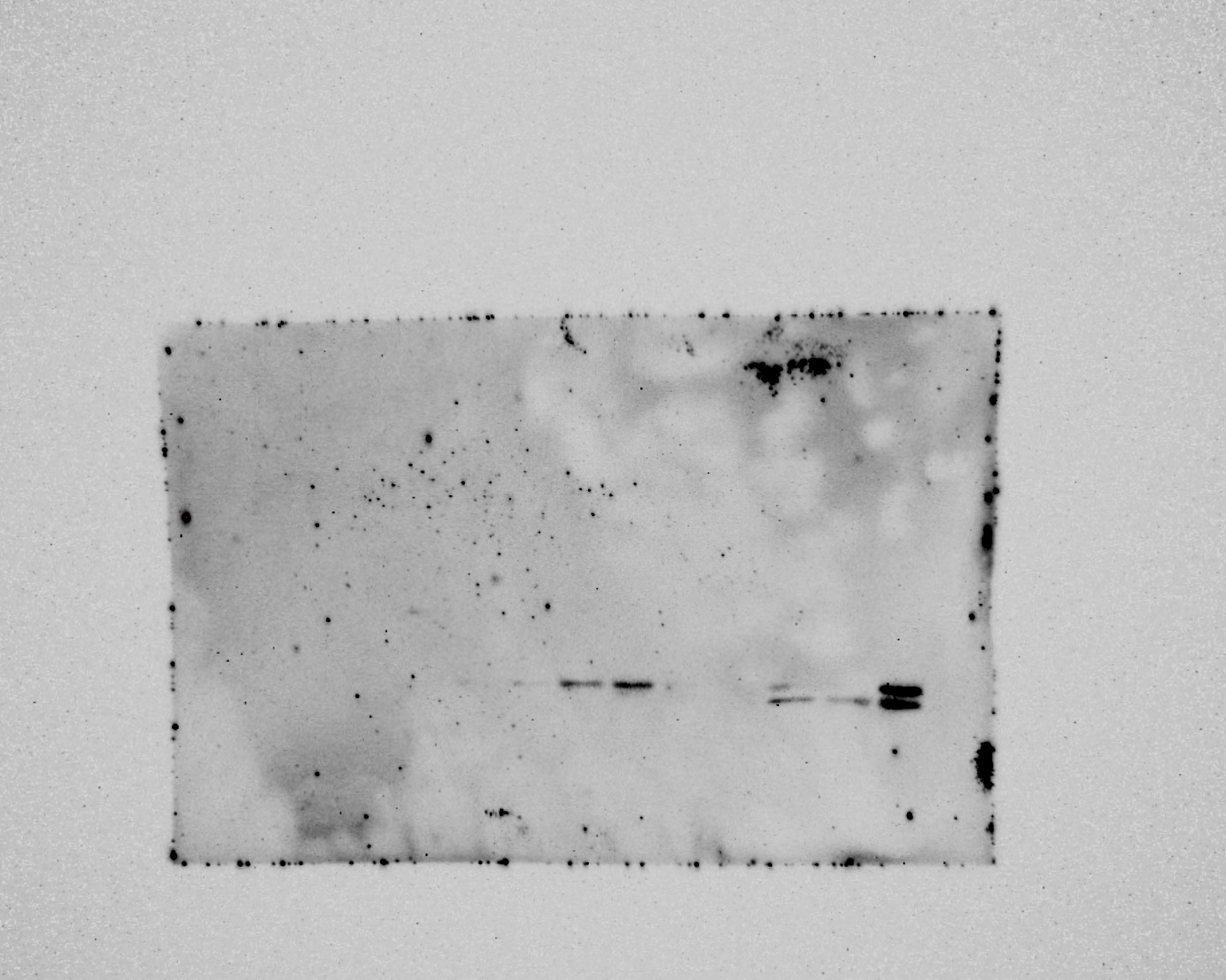

Supplement: Supplemental Information 3 — Non-modified Western-blots. [file peerj-06-5620-s003.zip › Fig2B IF3 raw data.tif]

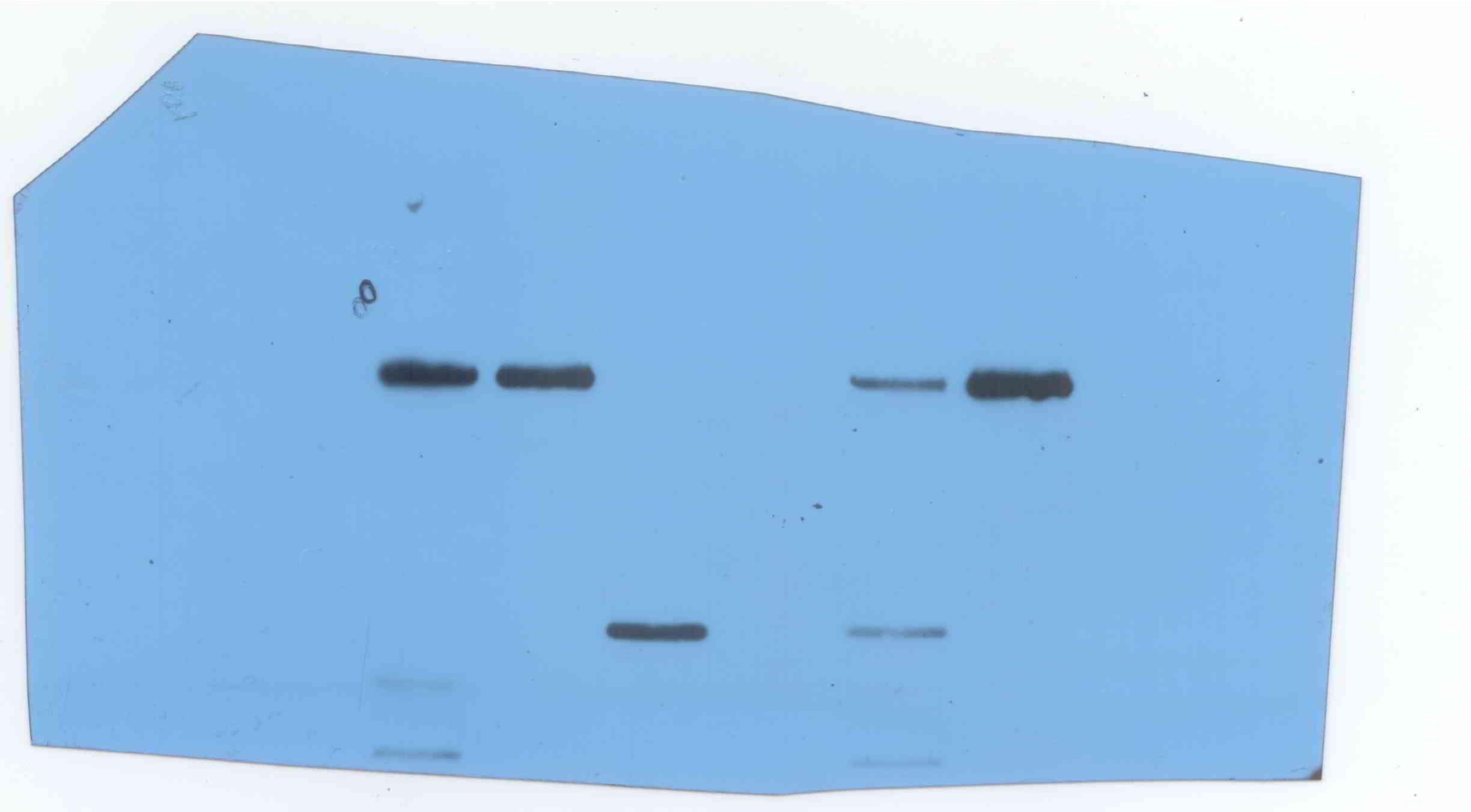

Supplement: Supplemental Information 7 — Non-modified Western-blot. [file peerj-06-5620-s007.jpg]

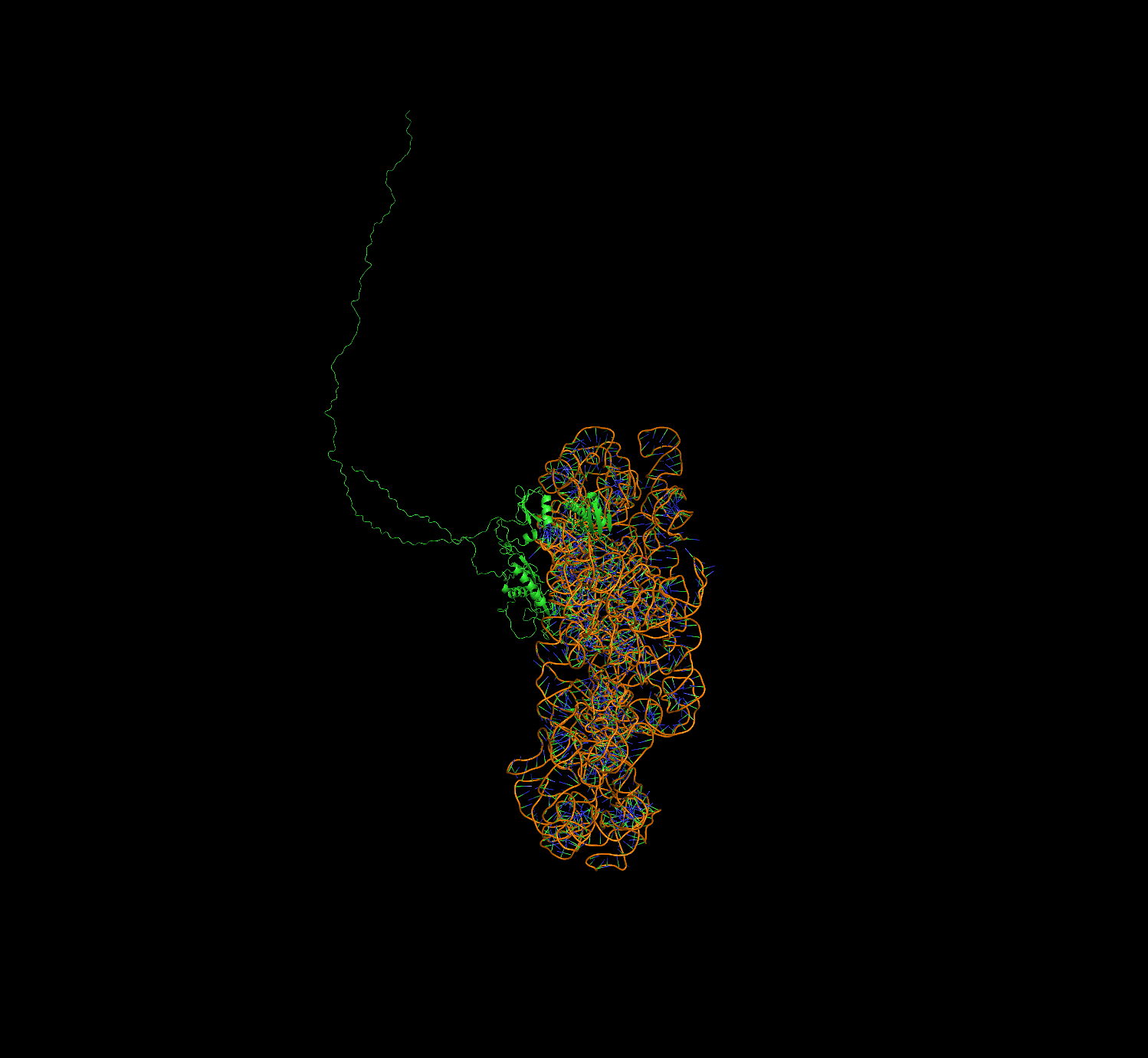

Supplement: Supplemental Information 8 — Results of molecular modeling. [file peerj-06-5620-s008.png]
