# Supplementary material for: 60S dynamic state of bacterial ribosome is fixed by yeast mitochondrial initiation factor 3
Source: PeerJ. 2018 Sep 17;6:e5620. doi: 10.7717/peerj.5620 (PMC6147165; doi:10.7717/peerj.5620)
Supplement: Supplemental Information 9 [file peerj-06-5620-s009.html]

modeller\_aim23-Kamensky slides


# Jupyter notebook to reporoduce modelling of aim32 complex with ribosome¶

At first step we import modeules from modeller 9.17

In [ ]:

```
import sys 
sys.path.append('/usr/lib/modeller9.17/modlib/')
sys.path.append('/usr/lib/modeller9.17/lib/x86_64-intel8/python2.5/')
import modeller 
import _modeller
import modeller.automodel 
import os
```

- Initilize modeller and allow use of HETATOM records from pdb file

In [13]:

```
env=modeller.environ()
env.io.hetatm=True
```

In [5]:

```
cd /home/localhdd/somework/kamensky
```

```
/home/localhdd/somework/kamensky
```

# Abinitio Folding of N-terminal extension with Rosetta¶

In [6]:

```
%%bash 
mkdir abinitio1
cd abinitio1
echo '>51773 someseq 
LKVPLSDVLSQKMLFLKSFRYFHCTKYFSRDNASSTTDIFRNAMKRKREL
ANLKEQSHGNVARNAAFPKEYIKRPKQVPRNATNRKKILITWSTGTDRAK
EAANSVVSE' > seq.fasta
echo '-in:file:fasta ./seq.fasta
-in:file:frag3 ./aat000_03_05.200_v1_3  
-in:file:frag9 ./aat000_09_05.200_v1_3  
-abinitio:relax                         
-nstruct 200000                              
-out:pdb_gz                                
-out:path abinitio-pdb ' > flags
/home/localhdd/rosetta_src_2017.08.59291_bundle/main/source/bin/AbinitioRelax.mpi.linuxgccrelease @flags >&log
```

## Select best structure from abinitio:¶

In [ ]:

```
%%bash 
awk '($1 == "SCORE:" && $2<100){print $31}' default.out  | sort -nk1 | head -n1 > topstructure
/home/localhdd/rosetta_src_2017.08.59291_bundle/main/source/bin/score_jd2.mpi.linuxgccrelease \
    -in:file:silent default.out -in:file:fullatom -out:pdb -out:file:fullatom \
    -in:file:tags $(cat topstructure)
```

- Prepare ribosome template , remove all chains from 5ME0.pdb except 16S RNA and IF3, saved to templ.pdb

In [21]:

```
alignm=modeller.alignment(env)
alignm.append(file='aim23.fasta', align_codes='all',alignment_format='FASTA')
mdl1 = modeller.model(env, file='templ.pdb', model_segment=('FIRST:'+'A', 'LAST:'+'Z'))
mdl2 = modeller.model(env, file='S_00029428_0001.pdb', model_segment=('FIRST:'+'A', 'LAST:'+'Z'))
alignm.append_model(mdl1, atom_files='templ.pdb', align_codes='rib')
alignm.append_model(mdl2, atom_files='S_00029428_0001.pdb', align_codes='ros')

! rm all_in_one.ali
alignm.salign()
s= alignm[1]
for i in range(len(alignm)):
    print "%s identical to wt in %4.1f perc" %(alignm[i].code,s.get_sequence_identity(alignm[i]))
alignm.write(file='all_in_one.ali', alignment_format='PIR')
```

- After initial aligment was done, it was replaced with aligment done with MUSCLE

In [ ]:

```
outstr = ''
from Bio import SeqIO
records = {}
for record in SeqIO.parse("all_in_one.ali", "pir"):
    print record.id
    records[record.id] = record.description

for record in SeqIO.parse("all_muscle.ali", "clustal"):
    if True : 
        outstr += '>P1;' + record.id + '\n'
        outstr += records[record.id] + '\n'
        outstr +=  record.seq + '*\n'

print len(outstr)
with open("full3.ali", "w") as out:
out.write(str(outstr))
```

## \* run modeller with modified automodel class to allow custom distance restrains ¶

In [ ]:

```
path = '/home/localhdd/somework/kamensky/'

class mymodel(modeller.automodel.automodel):
    megadistance = 10
    def special_restraints(self, aln):

        rsr = self.restraints
        at = self.atoms            
        d = self.megadistance
        rsr.add(modeller.forms.gaussian(group=modeller.physical.xy_distance,
            feature=modeller.features.distance(at['CA:61:A'],at['CA:293:A']),
            mean=d, stdev=d/10))
        rsr.add(modeller.forms.gaussian(group=modeller.physical.xy_distance,
            feature=modeller.features.distance(at['CA:61:A'],at['CA:236:A']),
            mean=d, stdev=d/10))
        rsr.add(modeller.forms.gaussian(group=modeller.physical.xy_distance,
            feature=modeller.features.distance(at['CA:61:A'],at['CA:184:A']),
            mean=d, stdev=d/10))

joblist = []
dist = [15.0,20.0,30.0,40.0]
for d in dist:
    
    env.libs.topology.clear()
    
    ! mkdir {d} ; cp full3.ali {d}; cp S_00029428_0001-b.pdb {d}; cp temp1.pdb {d}
    
            
    os.chdir(path + str(d))
    a = mymodel(env, alnfile='full4-r30.ali', knowns= ('rib','ros'), sequence = 'aim23-r30')
    a.name='mod'
    a.megadistance = d
    a.max_var_iterations = 1000
    a.repeat_optimization = 10
    a.md_level = modeller.automodel.refine.very_slow
    a.starting_model = 1
    a.ending_model = 10
    joblist.append(a)
    a.use_parallel_job(j)
    os.chdir(path)
    a.make()
```

# Select best models from modeller output¶

In [ ]:

```
%%bash
for i in "15.0" "20.0" "30.0" "40.0"; \
do grep 'OBJECTIVE FUNCTION:' ${i}/aim23*.pdb; \
done | sort -nk6
```

## Use FloppyTail to equlibrate C-terminal extension¶

In [ ]:

```
cat '''
-s aim23.best.pdb 

#fragments if you want them; only 3mers are used.
#-in:file:frag3 complex_readytail_final_nozn_fragset3mer.gz #frags are too big to include in the demo

-FloppyTail:flexible_start_resnum 320
-FloppyTail:flexible_stop_resnum  355
-FloppyTail:flexible_chain A
-FloppyTail:short_tail:short_tail_off 0
-FloppyTail:short_tail:short_tail_fraction 1.0

-FloppyTail:publication true

-FloppyTail:refine_repack_cycles 10

#low-end production numbers
-FloppyTail:perturb_cycles 15000
-FloppyTail:refine_cycles 10
-nstruct 5 
''' > floppyoptions
/home/localhdd/rosetta_src_2017.08.59291_bundle/main/source/bin/FloppyTail.mpi.linuxgccrelease @floppyoptions
```
